# Alternative dominance of the parental genomes in hybrid cells generated through the fusion of mouse embryonic stem cells with fibroblasts

**DOI:** 10.1038/s41598-017-18352-4

**Published:** 2017-12-22

**Authors:** Natalia M. Matveeva, Veniamin S. Fishman, Irina S. Zakharova, Alexander I. Shevchenko, Inna E. Pristyazhnyuk, Aleksei G. Menzorov, Oleg L. Serov

**Affiliations:** 1 Institute of Cytology and Genetics, Novosibirsk, 630090 Russia; 2 Novosibirsk State University, Novosibirsk, 630090 Russia; 3 E.N. Meshalkin National Medical Research Centre, Ministry of Health Care of Russian Federation, Novosibirsk, 630055 Russia; 4 Institute of Chemical Biology and Fundamental Medicine, Novosibirsk, 630090 Russia

## Abstract

For the first time, two types of hybrid cells with embryonic stem (ES) cell-like and fibroblast-like phenotypes were produced through the fusion of mouse ES cells with fibroblasts. Transcriptome analysis of 2,848 genes differentially expressed in the parental cells demonstrated that 34–43% of these genes are expressed in hybrid cells, consistent with their phenotypes; 25–29% of these genes display intermediate levels of expression, and 12–16% of these genes maintained expression at the parental cell level, inconsistent with the phenotype of the hybrid cell. Approximately 20% of the analyzed genes displayed unexpected expression patterns that differ from both parents. An unusual phenomenon was observed, namely, the illegitimate activation of *Xist* expression and the inactivation of one of two X-chromosomes in the near-tetraploid fibroblast-like hybrid cells, whereas both Xs were active before and after *in vitro* differentiation of the ES cell-like hybrid cells. These results and previous data obtained on heterokaryons suggest that the appearance of hybrid cells with a fibroblast-like phenotype reflects the reprogramming, rather than the induced differentiation, of the ES cell genome under the influence of a somatic partner.

## Introduction

Cell fusion with embryonic stem (ES) cells is a powerful tool for restoring pluripotency in somatic cells^1–5^. Hybrid cells obtained through the fusion of ES and somatic cells, as a rule, show characteristics of ES cells, including a capacity to generate chimeric embryos and even chimeric adult animals^1,6–11^. These data suggest the dominance of the ES cell genome over the somatic genome in diploid ES/diploid somatic cell hybrids. Previously, we observed two alternative phenotypes among heterokaryons produced through the fusion of mouse diploid ES cells with diploid fibroblasts^12^. One type of heterokaryons showed a fibroblast-like phenotype and expressed the typical fibroblast markers collagen type I and fibronectin but was negative for the pluripotent cell markers, Oct4 and Nanog. Another type of heterokaryons showed an ES cell-like phenotype and was positive for Oct4 and Nanog but negative for collagen type I, fibronectin and lamin A/C^12^. In addition, the last type of heterokaryons displayed signs of reactivation of the previously inactive X-chromosome. Importantly, hybrid cells, which appeared during the first 2–4 days after cell fusion, also displayed either ES cell-like or fibroblast-like phenotypes. However, the fates of these two types of hybrid cells were different: the ES cell-like hybrid cells formed colonies at 4–6 days, whereas the fibroblast-like hybrid cells grew as single cells and were unable to form colonies similar to mouse primary fibroblasts. Unfortunately, we were unable to determine chromosome composition or establish a ratio of the parental genomes in the fibroblast-like hybrid cells, reflecting their limited proliferating potential. This point is very important because after the fusion of ES cells and fibroblasts, hexaploid hybrid cells with 1:2 parental genome ratios are often formed, and the partner that introduces two copies of the genome ultimately defines the hybrid cell phenotype. Consistently, in a previous study, we demonstrated that the fusion of mouse tetraploid fibroblasts with diploid mouse ES cells generated hybrid cells with a fibroblast-like phenotype only^13^. Hence, we cannot exclude the likelihood that hybrid cells with fibroblast-like phenotypes were formed from the fusion of two fibroblasts and one ES cell.

This article is dedicated to the detailed characterization of a set of ES cell-like and fibroblast-like hybrid cells obtained through the fusion of mouse ES cells with m5S fibroblasts as a somatic partner. Both types of hybrid cells had stable near-tetraploid karyotypes and a ratio of the parental genomes close to 1:1. The m5S is a unique mouse fibroblast cell line with stable near-diploid karyotype capable of unlimited proliferation and clonogenicity^14^. We performed transcriptome RNA-seq analysis of both types of hybrid cells and discriminated the expression of 2,848 genes of both parental genomes. The transcriptome analysis revealed that although the sets of genes involved in the establishment of both phenotypes of hybrid cells were different, both types of hybrid cells had similar ratios of activated or silenced genes and genes with intermediate and “novel” expression. These data and previous our data^12^ obtained on heterokaryons suggest that the observed alternative manifestation of the parental genomes in two types of hybrid cells reflects the bidirectional reprogramming of the parental genomes.

## Results

### Characterization of ES cell-fibroblast hybrid cells with alternative manifestation of the parental genomes

In the first experiment, we used tau-GFP ES cells cultured in standard ES cell medium without 2i (PD0325901 and CHIR99021), and after fusion with m5S fibroblasts, we observed the formation of 50 primary HAT- and puromycin-resistant colonies: 15 colonies with an ES cell-like phenotype and 35 colonies with a fibroblast-like phenotype. In the second experiment, ES cells were cultured in the presence of 2i, and after fusion with m5S fibroblasts, 35 primary colonies with ES cell-like phenotypes and 148 colonies with fibroblast-like phenotypes were identified. In the third experiment, both ES cells and hybrid cells obtained after fusion were cultured in medium supplemented with 2i until harvest, and we observed hybrid cells with alternative phenotypes (89 primary ES cell-like colonies and 99 fibroblast-like colonies). These results suggest that the presence or absence of 2i in ES cell medium prior to cell fusion does not affect the prevalence of primary colonies with fibroblast-like phenotypes over ES cell-like phenotypes.

**Figure 1:**
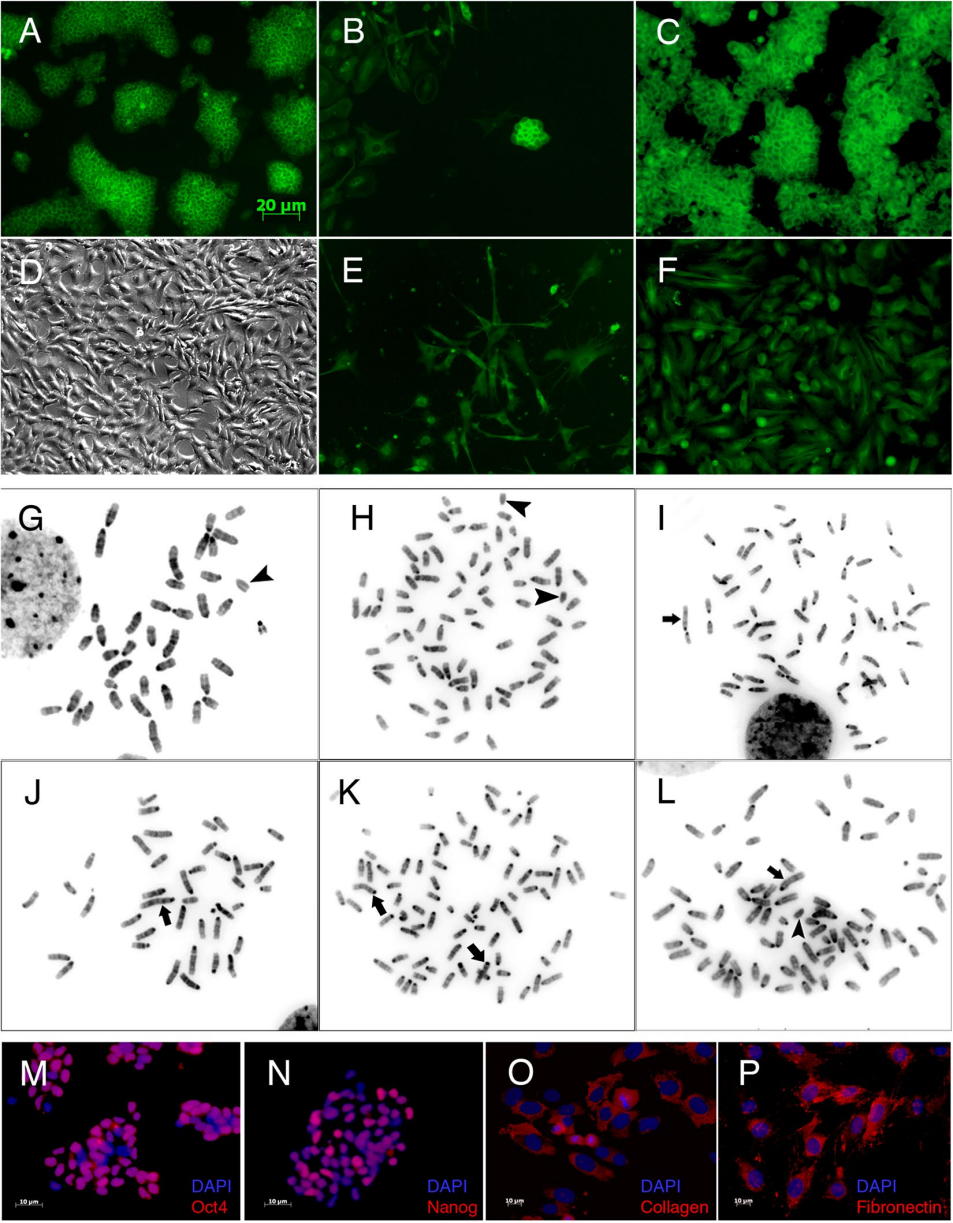
Phenotypes of parental and hybrid cells: (**A**) A tau-GFP ES cell line; (**B**) An ES cell-like colony of hybrid cells at 7 d after cell fusion; (**C**) Hybrid cell clone *tme14* with ES-like phenotype, 14 d after fusion; (**D**) A m5S cell line; (**E**) A colony of GFP-positive fibroblast-like hybrid cells at 7 d after fusion; (**F**) GFP-positive hybrid cell clone *tmf5* with a fibroblast-like phenotype at 21 d after fusion. (**G**,**J**) Metaphase spreads of tau-GFP ES cells and m5S cells with near-diploid chromosome sets, respectively. Arrowheads show the Y-chromosome, and arrows show a marker chromosome in m5S cells. (**H**,**K**) Metaphase spreads of tetraploid tau-GPF4N and tetraploid m5S4N8 cells, respectively. (**I**,**L**) Metaphase spreads of *tme13* and *tmf2* hybrid cell clones with near-tetraploid chromosome sets, respectively. (**M**,**N**) IF staining of hybrid cells for ES cell specific markers, Oct4 and Nanog, respectively. The presence of Oct4 (red) is shown in the nuclei of *tme17* hybrid cells, and the presence of Nanog (red) is shown in the nuclei of *tme14* hybrid cells. (**O**,**P**) IF staining of hybrid cells for the fibroblast markers collagen type I and fibronectin, respectively. The presence of collagen (red) is shown in the cytoplasm of *tmf1* cells, and the presence of fibronectin (red) is shown in the *tmf5* cells. Cell nuclei are stained with DAPI (blue).

Figure 1A,D illustrates the morphology of tau-GFP ES cells and m5S cells, respectively, and both types of hybrid cells at the early (B,E) and late stages (C,F) of colony formation. Both types of hybrid cells showed clearly different primary colony morphologies: ES cell-like cells (B,C) tended to form compact cell groups of tightly contiguous cells, whereas fibroblast-like cells (E,F) formed colonies of non-contacting cells. Primary hybrid cell clones with ES-like and fibroblast-like morphologies were designated *tme* and *tmf* series, respectively.

Figure 1M,N shows the results of the immunofluorescent (IF) analysis for the key markers of pluripotency, Oct4 and Nanog, in ES-like hybrid cells, respectively, and Fig. 1O,P demonstrates IF staining for the fibroblast-specific markers, collagen type I and fibronectin, respectively, in fibroblast-like hybrid cells. All cells with ES cell-like phenotypes were positive for Oct4 (Fig. 1M) and Nanog (Fig. 1N), and the majority of hybrid cells with fibroblast-like phenotypes were positive for collagen (Fig. 1O) and fibronectin (Fig. 1P).

### Identification of hybrid clones with near-tetraploid karyotypes and equal ratios of parental genomes

We performed a cytogenetic analysis of 30 hybrid clones of both types. Only 3 of 15 *tme* hybrid cell clones (*tme13*, *tme14*, and *tme17*) contained a near-tetraploid chromosome set. These clones contained 75%, 83%, and 92% cells with 74–78 chromosomes, respectively. All cells of these clones contained a single marker chromosome derived from m5S (Fig. 1I). Other clones of this series had near-hexaploid chromosome sets.

Among 15 clones of the *tmf* series, only 3 clones (*tmf1*, *tmf2*, and *tmf5)* contained near-tetraploid chromosome complements. These clones contained marker chromosomes derived from m5S cells and the Y-chromosome from tau-GFP (Fig. 1L). The numbers of chromosomes varied from 76 to 80 in most of the examined cells of these clones. Other clones of the *tmf* series also had near-hexaploid chromosome sets containing two marker chromosomes derived from m5S.

Three hybrid clones of *tme* series, *tme13*, *tme14*, and *tme17*, and three clones of the *tmf* series, *tmf1*, *tmf2*, and *tmf5*, with near-tetraploid chromosome composition were selected for further analysis. Microsatellite analysis demonstrated that these clones contained both parental allelic variants localized on chromosomes 13, 14, 15, 17, 18, and 19. These clones showed stable near-tetraploid karyotypes during prolonged cultivation.

Reflecting the different genetic background of parental cell lines, we detected single-nucleotide polymorphisms (SNPs) that distinguish parental genomes through transcriptome analysis (see below). We identified 27,962 SNPs distinguishing the parental cell lines derived from 129 and ICR mice. SNP analysis of hybrid clones *tmf2, tmf5, tme14* and *tme17* demonstrated that the vast majority of transcripts (from 94.8% to 97.3%) contained SNPs that originated from both parental alleles (Table 1).

**Table 1:** Number of SNPs identified in hybrid clones of the *tme* and *tmf* series.

Clone	Number of genes, containing SNPs		
	Only from one of the parental genomes	From both parental genomes	Some SNPs from one, and some from both parental genomes
*tme14*	34 (1.1%)	2,888 (96.8%)	62 (2.1%)
*tme17*	38 (1.2%)	2,985 (97.3%)	45 (1.5%)
*tmf2*	61 (2.0%)	2,892 (96.5%)	44 (1.5%)
*tmf5*	93 (3.1%)	2,874 (94.8%)	64 (2.1%)

Thus, the results of cytogenetic, microsatellite, and SNP analyses indicate that the selected clones, *tmf2*, *tmf5, tme14*, and *tme17*, showed stable karyotypes with parental genome ratios close to 1:1.

Since both types of hybrid cells had near-tetraploid karyotypes, we identified and isolated tetraploid cells from populations of both parental cells as relevant controls in transcriptome analysis. Spontaneous tetraploidization is a phenomenon observed in cell cultures, including ES and somatic cells. In contrast to induced polyploid cells, for example through cell fusion, spontaneously induced tetraploid cells with a stable 4 N karyotype were observed during prolonged cultivation. The cytogenetic analysis of 30 subclones derived from individual cells of diploid tau-GFP ES cells revealed two clones possessing tetraploid chromosome sets, and one of these subclones, tau-GFP4N, was used for further analysis (Fig. 1H).

In addition, we isolated cells with tetraploid chromosome set from a diploid m5S cell population through preliminary sorting according to size, followed by subcloning. Cytogenetic analysis of the subclones revealed a clone with a tetraploid karyotype (Fig. 1K), and this clone was designated m5S4N8 and used for further transcriptome RNAseq analysis.

### Transcriptome analysis of *tme* and *tmf* hybrid cells

**Figure 2:**
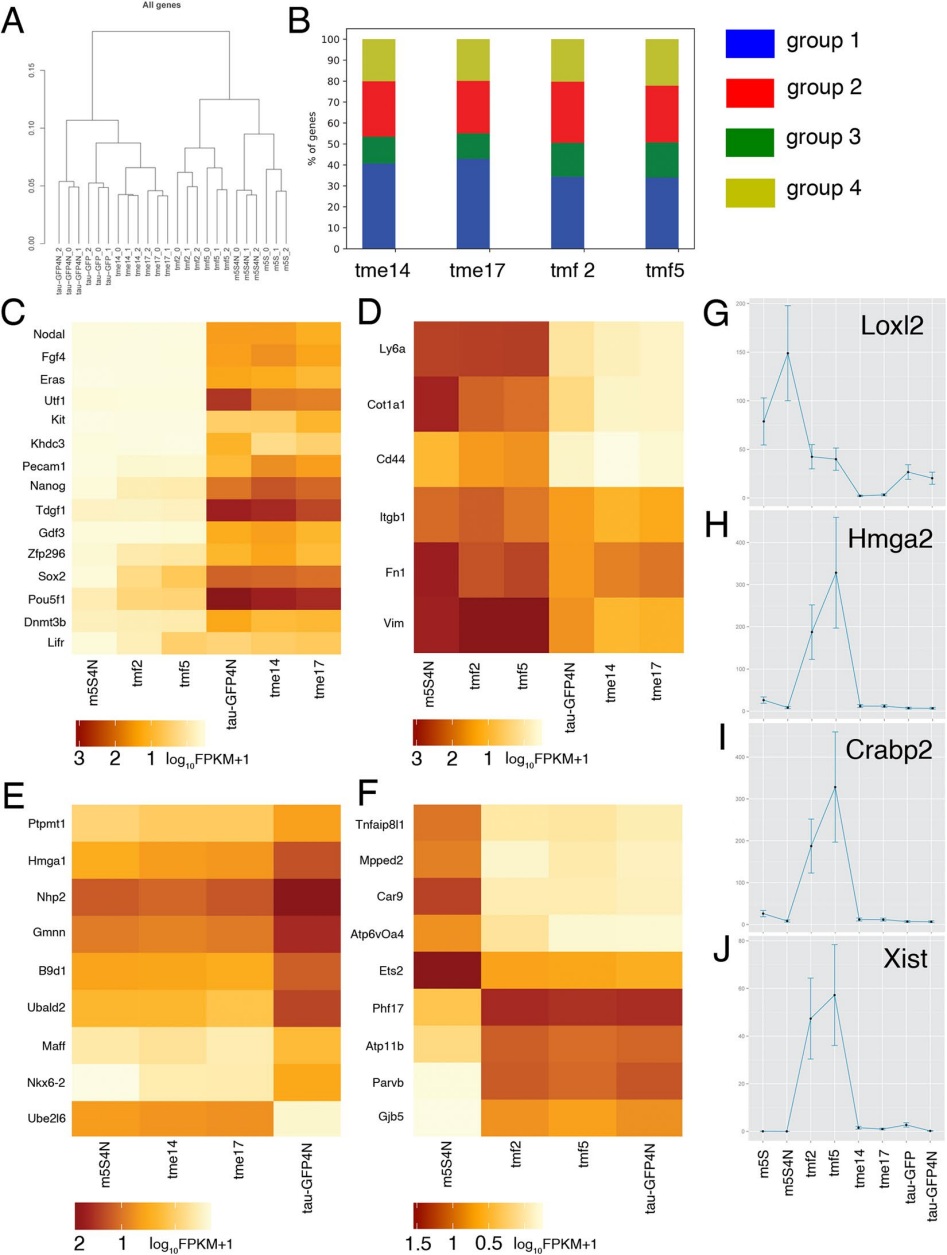
RNA-sequencing based transcriptome analyses of hybrid cells. (**A**) Hierarchical clusters based on the analysis of 23,361 genes expressed in tau-GPF4N, m5S4N8, tau-GFP ES cells, m5S cells and both *tme* and *tmf* hybrid cell types. (**B**) Bar-plot showing proportion of genes belonging to groups 1–4 in *tme* and *tmf* clones (see Results for group definitions). (**C**,**D**) Heat map of the gene expression profiles of the marker genes specific for ES cells (**C**) and fibroblasts (**D**) in hybrid cells of the *tme* and *tmf* series, tau-GPF4N and m5S4N8 cells. (**E**,**F**) Heat map presentation of gene-expression profiles of some genes keeping expression in *tme* hybrid cells similar to that in m5S4N cells (**E**) and genes maintaining expression in *tmf* hybrid cells similar to that in tau-GFP4N cells (**F**). For (**C**–**F**) the colors represent log_10_FPKM + 1 values according to the scale shown at the bottom. Expression levels of the *Loxl2* (**G**), the *Hmga2* (**H**), the *Crabp2* (**I**) and the *Xist* (**J**) genes in hybrid cell clones and parental cells. The Y-axis represents FPKM values for corresponding genes.

Two *tme* hybrid clones (*tme14* and *tme17*) and two *tmf* clones (*tmf2* and *tmf5*) were selected for RNA sequencing-based transcriptome analysis. Both diploid parental cells, tau-GFP ES cells and m5S cells, and their two tetraploid derivatives, tau-GFP4N and m5S4N8 cells, were subjected to RNA sequencing. The expression profiles of 23,361 genes were determined in the hybrid clones and parental cell lines. The subsequent clustering of the gene expression profiles demonstrated high similarity between the hybrid clones *tme14* and *tme17*, showing an ES cell-like phenotype, and diploid and tetraploid ES cells, as well as between the hybrid clones *tmf2* and *tmf5*, showing a fibroblast-like phenotype, and diploid m5S and tetraploid m5S4N8 cells (Fig. 2A). Interestingly, hybrid clones with the same phenotypes were more similar to each other than to the parental cells (Fig. 2A).

We performed detailed comparisons of the gene expression profiles of hybrid clones of both types and parental cells. For this comparison, we examined the gene expression levels of tetraploid tau-GFP4N and m5S4N8 cells to eliminate the influence of the different ploidy statuses of hybrid cells possessing near-tetraploid chromosome sets and both diploid parental cell lines.

Several gene pools were identified among the 23,361 analyzed genes: a) 14,794 genes expressed at a low level (<10 FPKM) in both parental cell lines and all hybrid clones; b) 2,274 genes expressed at the same level in both parental cell lines; c) 2,988 genes with minor (less than two times) differences between expression levels in tau-GFP4N and m5S4N8 cells; and d) 2,848 differentially expressed genes (more than two times) in tau-GFP4N and m5S4N8 cells. Significant differences in the expression levels of these 2,848 genes reliably reflected differences between the phenotypes of the parental cells.

According to the transcriptome analysis, many differentially expressed genes (34–43% of the analyzed genes) showed expression consistent with the phenotypes of hybrid cells, *i.e*., these genes were expressed in fibroblast-like *tmf* clones at the level of m5S4N8 cells and in ES cell-like *tme* clones at the level of tau-GFP4N cells. This gene pool (group 1; Fig. 2B; Table 2) includes genes either silenced or activated during the establishment of ES cell-like or fibroblast-like phenotypes in hybrid cell clones. The expression profiles of 15 pluripotency-associated genes and 6 fibroblast marker genes in m5S4N8 cells, tau-GFP4N cells and hybrid clones of both series are represented in Fig. 2C and D. The gene-expression profiles of *tme* series hybrid clones were remarkably similar to those of tau-GFP4N cells, whereas the gene expression profiles of *tmf* series hybrid clones were similar to those of m5S4N8 cells. We examined the SNPs to identify the activation of the m5S-derived alleles of pluripotency-associated genes, such as *Oct4* and *Nanog*, in ES cell-like hybrid cells and the tau-GFP-derived alleles of collagen and fibronectin in fibroblast-like hybrid cells.

**Table 2:** Classification of genes based on expression in tau-GFP4N cells, m5S4N8 fibroblasts and both types of hybrid clones.

Name of hybrid clone	Subgroups	Number of genes*	Percentage of all genes with 2-fold differences in expression in tau-GFP4N and m5S4N8 cells and tau-GFP ES and m5S cells (shown in brackets)
*tme14*	1^st^	933 (1477)	40.7 (57.3)
*tme14*	2^nd^	607 (666)	26.5 (25.8)
*tme14*	3^rd^	293 (199)	12.8 (7.7)
*tme14*	4^th^	460 (235)	20.1 (9.1)
*tme14*	Low expression (FPKM < 10)**	555 (609)	—
*tme17*	1^st^	1008 (1288)	42.8 (50.0)
*tme17*	2^nd^	589 (749)	25.1 (32.9)
*tme17*	3^rd^	287 (267)	12.2 (10,3)
*tme17*	4^th^	467 (269)	19,9 (10.4)
*tme17*	Low expression (FPKM < 10)**	467 (613)	—
*tmf2*	1^st^	828 (1341)	34.3 (50.0)
*tmf2*	2^nd^	704 (826)	29.2 (30.8)
*tmf2*	3^rd^	391 (260)	16.2 (9.7)
*tmf2*	4^th^	488 (253)	20.2 (9.4)
*tmf2*	Low expression (FPKM < 10)**	437 (506)	—
*tmf5*	1^st^	822 (1313)	34.0 (48.8)
*tmf5*	2^nd^	654 (786)	27.0 (29.1)
*tmf5*	3^rd^	406 (265)	16.8 (9.8)
*tmf5*	4^th^	537 (323)	22.2 (12.0)
*tmf5*	Low expression (FPKM < 10)**	429 (499)	—

*Gene numbers indicate comparisons with tetraploid tau-GFP4N and m5S4N8 cells, whereas brackets indicate comparisons with diploid tau-GFP and m5S cells. **During the analysis of individual clones, only genes expressed at high (>10 FPKM) levels were considered. Genes expressed at low levels (e.g., <10 FPKM) in both parental cells were only considered for hybrid clones where the expression of this gene was above the threshold value (10 FPKM).

Approximately 25–29% of genes (group 2; Fig. 2B; Table 2) showed “intermediate” levels of expression in hybrid clones compared to both parental cells. Figure 2G illustrates the intermediate expression of the parental alleles at the *Loxl2* locus (gene for lysyl oxidase homolog 2) with respect to the parental cells in fibroblast-like hybrid cells but not in ES-like hybrid clones.

Approximately 12–17% of genes (group 3) maintained expression in hybrid cells at a level similar to that of one of the parental cell lines (Fig. 2B; Table 2). Figure 2E illustrates the obvious differences in the expression of nine genes in m5S4N8 and tau-GFP4N cells. However, as shown in Fig. 2E, the expression of these genes in hybrid cell clones with an ES-cell phenotype remains at a level similar to that in m5S4N8 cells, and in contrast, the expression of some genes in hybrid clones of the *tmf* series was maintained at the level of tau-GFP4N8 cells, despite their fibroblast-like phenotype (Fig. 2F). Thus, the expression of these genes, identified in all analyzed hybrid clones, is inconsistent with the phenotype of hybrid cells.

The transcriptome analysis revealed an unexpected pool of genes (20–22% of the analyzed genes, group 4; Fig. 2B; Table 2) expressed in hybrid cells at levels significantly different from both parental cell lines, with expression levels either above or below the expression levels in both parental cells. The expression of these genes can be designated “novel”, *i.e*., their expression is characteristic of hybrid cells (Fig. 2B; Table 2). Here should be pertinent to note that both parental alleles of the “novel” genes contribute to the observed expression pattern. This conclusion was based on the SNP analysis showing that most of SNPs distinguishing parental cells were equally represented in transcripts of genes with the “novel” expression pattern (the data not shown).

Following we provide several examples of genes with the “novel” expression pattern. The *Loxl2* gene is expressed at a high level in m5S4N8 cells, a moderate level in tau-GFP4N cells, an intermediate level in *tmf2* and *tmf5* hybrid cells and is practically absent in *tme14* and *tme17* clones (Fig. 2G). Similarly, the expression of *Hmga2* and *Crabp2* genes was low in both parental cells and hybrid clones of the *tme* series, but dramatically increased in *tmf2* and *tmf5* clones (Fig. 2H,I). We have detected an unusual pattern of expression of the *Xist* locus (*the X-inactivation specific transcript)* in *tmf* hybrid cells but not in diploid and tetraploid parental cell lines (Fig. 2J). The *Xist* was not expressed either in m5S cells or tau-GFP ES cells because both cell types contained a single copy of the X chromosome. Also the *Xist* was not expressed in tetraploid m5S4N8 and tau-GFP4N containing each two copies of the X-chromosome. However, the expression of *Xist* was observed in *tmf* hybrid cells with a fibroblast-like phenotype but not in *tme* hybrid cells (Fig. 2J). The unexpected pattern of *Xist* expression prompted a more detailed analysis of the markers of X-chromosome inactivation in *tmf* hybrid cells.

### Signs of X-chromosome inactivation in hybrid cells of *tmf* series

**Figure 3:**
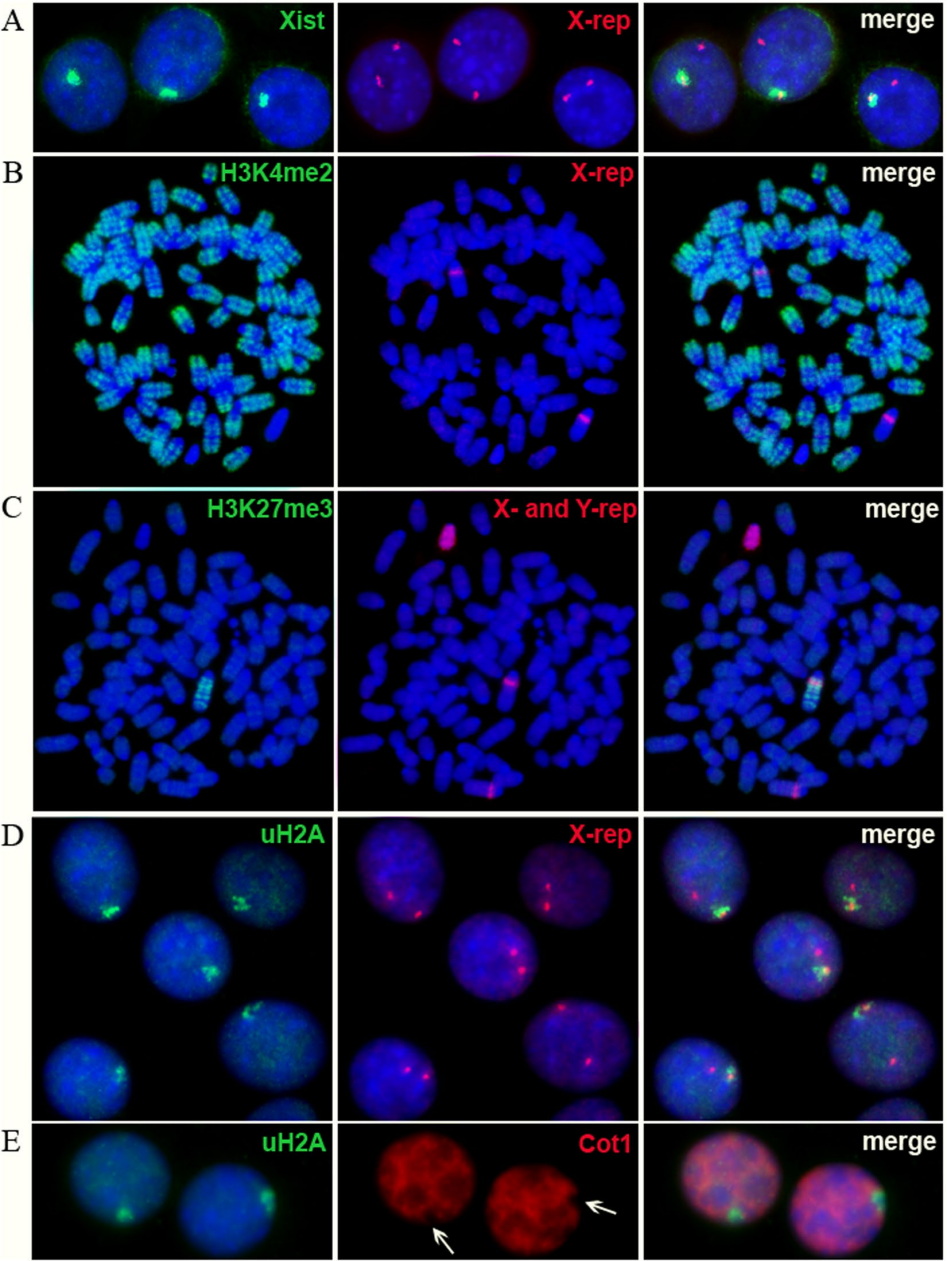
X-chromosome inactivation in the *tmf* series hybrid cells. (**A**) *Xist* RNA cloud (green) co-localizes with one of the two X-chromosomes, which were visualized by a repeat probe (red). (**B**) Exclusion of H3K4me2 (green) attributable to inactive chromatin from one of the two X-chromosomes (red). (**C**) Enrichment of an inactive X-chromosome with Н3K27me3 (green). X- and Y-chromosomes were recognized by X- and Y-specific repeats (both in red). (**D**) Cloud of ubiquitinated H2A (uH2A, green) co-localizes with one of the two X-chromosomes (red). (**E**) Nuclear region enriched with uH2A (green) after subsequent RNA FISH shows exclusion (indicated with arrows) of Cot1 DNA probe (red), which hybridizes with all unspliced gene transcripts within the nuclei.

**Figure 4:**
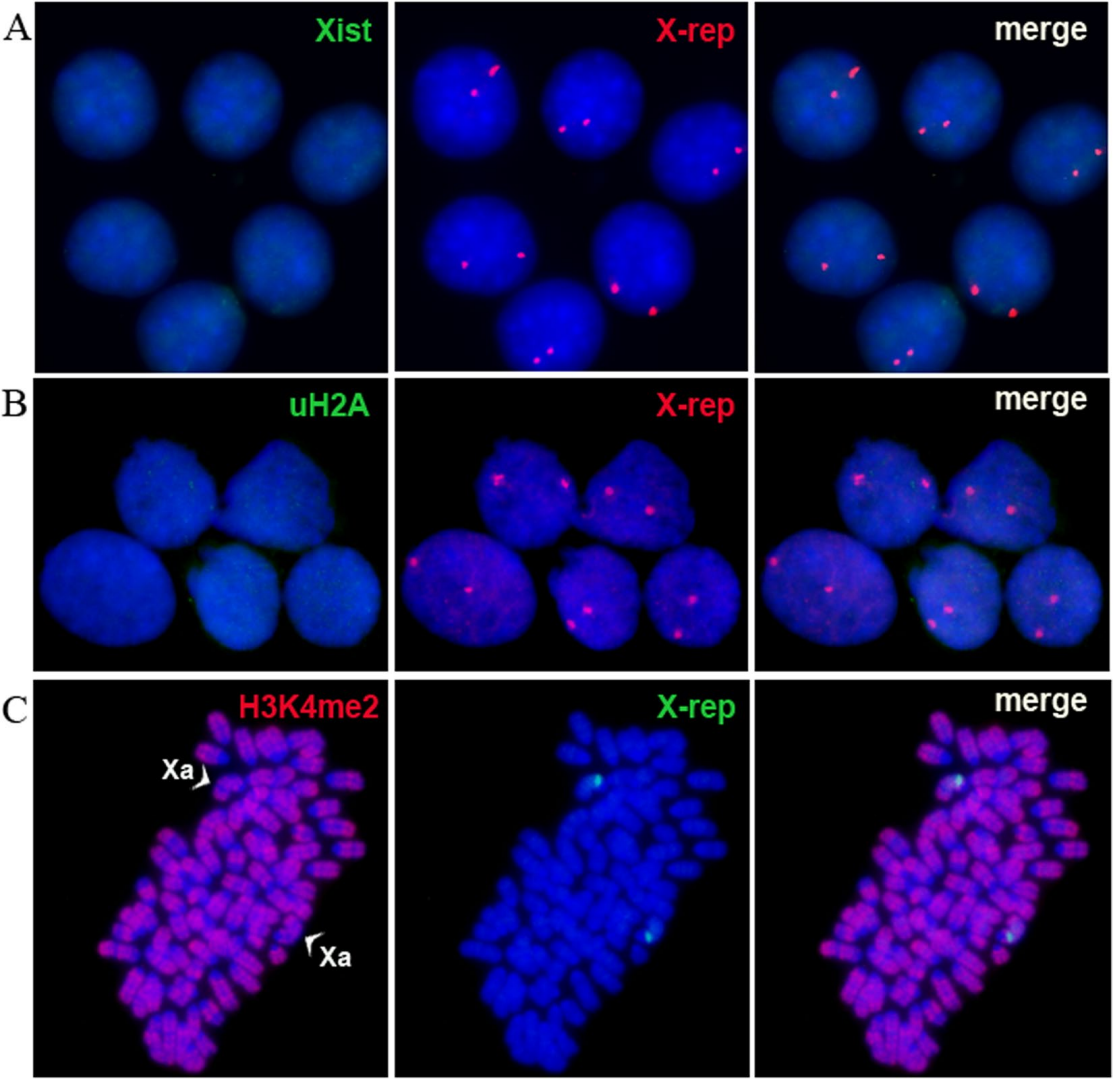
Tetraploid XXOO m5S4N8 cells maintain two active X-chromosomes. (**A**) No *Xist* RNA signal (green) on X-chromosomes (red) in tetraploid XXOO m5S4N8 cells. (**B**) No uH2A enrichment detected on X-chromosomes (red) in tetraploid XXOO m5S4N8 cells. (**C**) Both X-chromosomes (green) in tetraploid XXOO cells are positive for the active chromatin marker Н3K4me2 (red). Arrowheads point to active X chromosomes (Xa).

Combined RNA-DNA FISH analysis revealed that the nuclei of *tmf* cells were positive for *Xist* RNA, represented as a cloud associated with one of the two X-chromosomes (Fig. 3A). Moreover, one X-chromosome was negative for dimethylated H3K4, a marker of active chromatin, but enriched for repressive histone markers, such as trimethylated H3K27 and ubiquitinated H2A (Fig. 3B–D). In addition, the nuclear region containing inactive X-chromosome chromatin in sequential RNA FISH experiments was represented as Cot1 exclusion in *tmf* hybrid cells, also indicating the transcriptional repression of one of the X-chromosomes (Fig. 3E). These data unambiguously indicate the transcriptional repression of one of the X-chromosomes in *tmf* hybrid cells (Fig. 3A–E). In addition, similar analysis of active and inactive X-chromosome markers in tetraploid m5S4N8 cells demonstrated the absence of any signs of X-inactivation in these cells (Fig. 4A–C).

Importantly, SNP analysis of the RNA-sequencing transcriptome data demonstrated that both parental alleles of 65 genes localized on the X chromosome were expressed in *tmf* hybrid cells. Notably, none of these 65 genes escaped inactivation.

**Figure 5:**
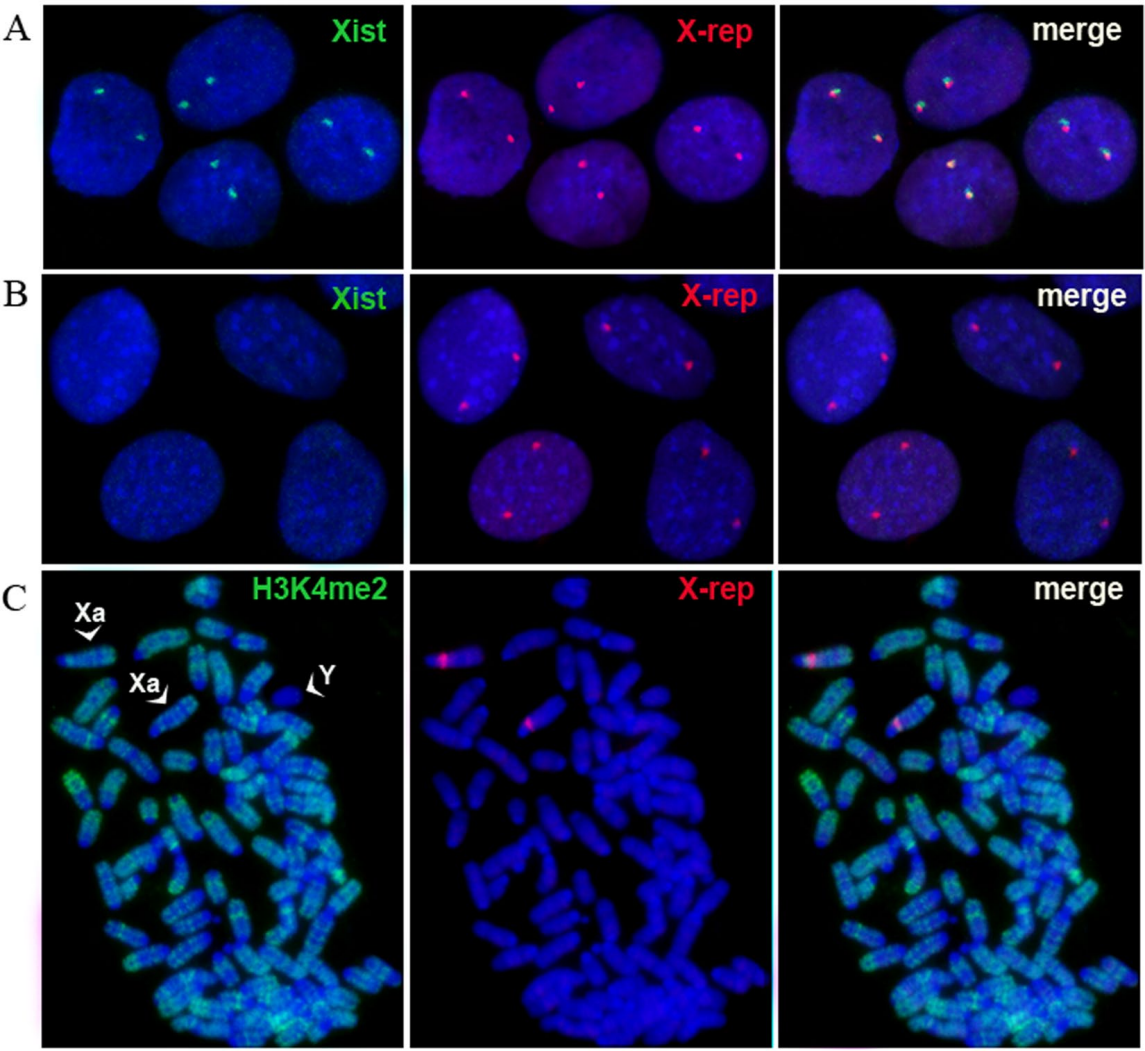
Tetraploid ES cell-like *tme* hybrid cells and their differentiated derivatives maintain two active X-chromosomes. (**A**) A pinpoint *Xist* signal (green) is revealed on each X-chromosome (red) in *tme* cells. (**B**) No *Xist* RNA signal (green) detected on X-chromosomes (red) in *tme* cells after 14 d of *in vitro* differentiation. (**C**) Both X-chromosomes (red) in differentiated *tme* cells are positive for the active chromatin marker Н3K4me2 (green). Arrowheads indicate active X (Xa) and Y-chromosomes.

Since both X-chromosomes were active in ES cell-like hybrid cells, a question naturally arises: how do the parental X-chromosomes behave in *tme* hybrid cells during *in vitro* differentiation? We performed RNA-DNA FISH analysis of the *tme* hybrid cells during *in vitro-*induced differentiation for 14 days. As shown in Fig. 5A, pinpoint signals of the labeled *Xist* probe over nuclei of the undifferentiated ES cell-like hybrid cells were observed. Previous studies have shown that these signals reflect the relationship between the *Xist* probe and its antisense counterpart *Tsix* ^15^. Importantly, these pinpoint signals disappeared after 10–14 days of *in vitro* differentiation of the *tme* hybrid cells (Fig. 5B), *i.e*., X-chromosomes positive for *Xist* transcripts were not identified. Moreover, both X-chromosomes were positive for dimethylated H3K4, a marker of active chromatin (Fig. 5C). Thus, hybrid clones of the *tme* series with an ES cell-like phenotype maintain both Х-chromosomes in an active state, both before and after *in vitro* differentiation, *i.e*., the behavior of two X-chromosomes was similar to that expected and characterized for tetraploid cells carrying two X-chromosomes.

## Discussion

The obtained data clearly indicated that the fusion of diploid ES cells and near-diploid fibroblast cells generates two types of hybrid cells with alternative manifestations of the parental genomes. The formation of hybrid cell colonies with a fibroblast-like phenotype and the well-known ES cell-like phenotype is feasible using clonogenic m5S fibroblasts as somatic partners^14^.

The prevalence of a number of primary fibroblast-like hybrid cell clones over ES cell-like clones is consistent with the results of previous studies on the appearance of two alternative phenotypes, ES cell-like and fibroblast-like phenotypes, among heterokaryons produced through the fusion of mouse diploid ES cells with diploid fibroblasts. In fact, within 20 h after fusion, most heterokaryons (up to 80%) showed a fibroblast-like phenotype, whereas approximately 20% of heterokaryons showed an ES-like phenotype, *i.e*., there was obvious prevalence in the formation of fibroblast-like over ES cell-like heterokaryons^12^.

We observed a high number of fibroblast-like hybrid cell clones in the presence and absence of 2i in ES cell medium. The presence of 2i in ES cell medium prevents differentiation^16^. Thus, a high amount of fibroblast-like hybrid cell clones was observed, even in the presence of 2i, suggesting that these clones result from the fusion of fibroblasts with undifferentiated ES cells.

We also proposed this hypothesis in a previous study regarding the dynamics of the DNA methylation patterns of the parental *Oct4* promoters in ES cell-fibroblast hybrid cells during the early stages of formation^12^. We demonstrated that the level of DNA methylation of the *Oct4* promoter derived from ES cell partner in the fibroblast-like hybrid cells was 2% at 48 hrs after fusion, whereas the DNA methylation of the fibroblast-derived allele was over 50% in this type of hybrid cells. However, 96 hrs after fusion, both parental alleles of the *Oct4* promoter showed similar levels of DNA methylation at approximately 50%. The presence of the unmethylated *Oct4* allele at the early stages of hybrid cell formation suggests that fibroblast-like hybrid cells originate from the fusion of undifferentiated ES cells with fibroblasts. In contrast, a much higher level of DNA methylation of the ES cell-derived *Oct4* promoter would be expected at the early stages after cell fusion if the differentiated ES cells were fused with fibroblasts.

Importantly, among numerous hybrid cell clones with alternative phenotypes, clones with a stable near-tetraploid karyotype were detected, and their parental chromosomes presumably represented equal proportions, based on cytogenetic, microsatellite, and SNP analyses.

Recently, similar cell fusion experiments were performed^11^ in which the authors generated hybrid cells through the fusion of the rat fibroblasts R1A with mouse ES cells. Although near-diploid R1A cells are immortalized and can be easily subcloned^17^, the hybrid cells generated in these experiments had an ES cell-like phenotype only^11^. Perhaps the hybrid cells with a fibroblast-like phenotype were not identified in these experiments because of differences in the growth rates of hybrid cells with alternative phenotypes. Moreover, since analysis at the early heterokaryon stage or during hybrid cell formation was not performed, the likelihood that hybrid cells with fibroblast-like phenotypes were present soon after cell fusion cannot be excluded.

The phenotypic similarities between the *tme* hybrid cells and ES cells and between the *tmf* hybrid cells and fibroblasts in the present study were confirmed by independent evidence, including the morphology of the cells, IF analysis of markers specific for ES cells and fibroblasts, hierarchical clustering of genome-wide expression profiles, and statistical analysis of individual gene expression.

The acquisition of an ES cell-like phenotype by hybrid cells is a well-known phenomenon based on the reprogramming of the fibroblast partner genome^3,8,10,18^, whereas the acquisition of a fibroblast-like phenotype can potentially be realized via either reprogramming of the ES cell genome or the induction of differentiation under the influence of the fibroblast genome after cell fusion.

In this context, it is important to consider a role for the different gene groups identified through transcriptome analysis in the establishment of alternative phenotypes of hybrid cells. The most numerous group 1 comprises silenced and activated genes that dramatically change expression during the hybrid cell clone formation, consistent with the phenotype of a hybrid cell. The phenomenon of gene silencing and activation is typical for somatic cell reprogramming after fusion with ES cells^3,4,8,19–21^. Undoubtedly, these genes are responsible for the establishment of the phenotype of hybrid cells. Similarly, the activation and silencing of specific genes underlies the reprogramming of somatic cell genomes based on the ectopic expression of defined transcription factors^21–27^.

According to the present transcriptome analysis, gene group 2 includes genes that are expressed at an intermediate level in both of types of hybrid cells with respect to parental cells. Ambrosi *et al*.^8^ previously described a similar gene group. Studies have shown that the level of gene expression in tetraploid cells differs from that in diploid cells in a nonlinear manner and often varies for individual genes^28^.

In contrast to gene groups 1 and 2, approximately 12–17% of the analyzed 2,848 genes (the gene group 3) demonstrate an expression profile inconsistent with expectations based on the phenotype of hybrid cells. A similar gene category, designated as non-reprogramming, was identified in induced pluripotent stem (iPS) cells generated via the ectopic expression of Yamanaka’s transcription factor cocktail in different somatic cells. Despite the acquisition of pluripotency, some genes maintained expression in iPS cells similar to that in targeted somatic cells, *i.e*., are not reprogrammed. The most convincing explanation for this phenomenon is epigenetic memory^29–31^. Interestingly, epigenetic memory, *i.e*., insensitivity to factors restoring pluripotency in the somatic genome, is characteristic of genes located close to centromeres and telomeres^31^. As described above, we did not observe preferable chromosome localizations of gene group 3 in both types of hybrid cells.

Group 4, comprising genes with “novel” expression, is the most enigmatic cell group characterized by a specific expression pattern exclusively observed in hybrid cells but not in parental cells. Similar category of genes was observed earlier, as clusters 1, 5 and 7, in ES cell-fibroblast hybrids with ES-like-phenotype^8^. This unusual expression may reflect mistakes in gene regulation. A striking example is the *Xist* expression, which is associated with illegitimate X-chromosome inactivation in *tmf* hybrid cells. Presumably, this mistake occurred in the chromosome counting mechanism, leading to the incorrect determination of the X-chromosome to autosomal sets ratio. Importantly, we did not observe any signs of X-chromosome inactivation during the *in vitro* differentiation of the ES-like hybrid cells in the present study.

A high percentage of genes with presumably incorrect expression, described in this and others studies^8^, is characteristic of hybrid cells obtained through the fusion of ES cells with somatic cells because there are no data showing a high percentage of similar types of gene expression in fully reprogrammed iPS cells^21–27^. Comparison of the various reprogramming technologies suggested that reprogramming induced through defined transcription factors requires at least two weeks and occurs through several consecutive stages^24–27^, whereas the reprogramming of the somatic genome after fusion with ES cells is initiated at the heterokaryon stage^12,32,33^ and completed after 4 days, evidenced by the *Oct4* promoter DNA demethylation of the somatic genome^12^. The timing of cell fusion-induced reprogramming resembles somatic cell transdifferentiation^34^ more than iPS cell generation. Differences in the timing of these processes may result in the incorrect expression of “novel” genes in hybrid cells and the absence or paucity of such genes in fully reprogrammed iPS cells.

Despite the high percentage of genes detected in groups 3 and 4 in the present study, previous studies have shown a high level of pluripotency in ES cell-somatic hybrid cells^1–3,7,9,35^. Hybrid cells have the capacity to generate chimeric embryos and even chimeric animals with a high contribution of tetraploid hybrid cells^10,36^. Since there is no mechanism for generating offspring from chimeras, due to the high ploidy of hybrid cells, the actual level of pluripotency in ES cell-fibroblast hybrid cells remains unknown. However, considering that a high percentage of genes are not involved or are incorrectly involved in restoring pluripotency in ES cell-like hybrid cells, the state of hybrid cells may reflect the incomplete reprogramming observed during the early stages of iPS cell formation^24–26^.

Now there is a reason to consider possible ways of establishment of fibroblast-like phenotype in ES cell-fibroblast hybrids. An important question is, whether establishment of the fibroblast-like phenotype is due to differentiation of the ES-cell genome under the influence of the fibroblast genome? Below we provide several arguments against this hypothesis.

Firstly, the obtained data demonstrated reliably that direct inhibition of ES cell differentiation by 2i^16^ before and even after fusion had no effect on obvious prevalence of the formation of fibroblast-like over ES cell-like hybrid cells. This is reliable argument against significant role of differentiation of ES cells in establishment of hybrid cells with fibroblast-like phenotype.

Secondly, according to our previous work^12^, within 20 h after fusion of ES cells and fibroblasts, most heterokaryons (up to 80%) have a fibroblast-like phenotype, being positive for typical fibroblast markers (collagen type I, fibronectin, lamin A/C) and me3H3K27 chromatin modification associated with the inactive X chromosome and being negative for Oct4 and Nanog^12^. Thus, signs of fibroblast differentiation appear before 1^st^ cell division whereas *in vitro* differentiation of ES cells requires up two weeks.

Thirdly, unusual expression of “novel” genes is presumably a result of mistake in gene regulation. This assumption is confirmed by the high percentage of genes expressed in hybrid cells, inconsistent with their phenotypes and unexpected in the case of ES cell differentiation. The most striking example for this is the *Xist* expression and other signs of X-chromosome inactivation in fibroblast-like hybrid cells presumably due to the incorrect counting of the X-chromosome to autosomal sets ratio. Indeed, we did not observed any signs of X-chromosome inactivation during the *in vitro* differentiation of the ES-like hybrid cells having the same ratio of the X-chromosomes and autosomes.

In the fourth, similarity of proportions of genes belonging to different categories in ES-cell-like and fibroblast-like hybrid cells is amazing if to remember that the sets of genes involved in establishment of their phenotypes are different.

Thus, the appearance of hybrid cells with a fibroblast-like phenotype reflects the reprogramming rather than the induced differentiation of the ES cell genome under the influence of the somatic genome. Of course, we cannot completely exclude possibility of ES cell genome differentiation during establishment of hybrid cells with fibroblast-like phenotype. However, taking in mind aforementioned arguments against this hypothesis, we believe that the observed alternative dominance of the parental genomes in ES cell-fibroblast cell hybrids is a result of bidirectional reprogramming.

Both alternative directions in establishment of both phenotypes are realized according to the “all or none” principle based on alternative phenotypes and RNA-sequencing data. The question remains: how is the choice of reprogramming direction realized? The first signs of unidirectional^32,33,37–40^ and bidirectional reprogramming^12^ are manifested at the heterokaryon stage within the first day after the fusion of ES cells with somatic cells. Importantly, parental cells are often at different stages of the cell cycle at the moment of cell fusion, and as a result, these cells asynchronously enter the first mitosis during hybrid cell formation. In addition, some data exist concerning the effect of cell cycle stages of fused partners on reprogramming^41^. For example, the fusion of ES cells with serum-starved quiescent fibroblasts showed a 38.6-fold higher efficiency of hybrid cell colony formation than ES cells fused with proliferating fibroblasts^18^. The gene expression profiles of the parental genomes are different and maintained through different sets of transcription factors. Asynchrony in the cell cycles of parental cells during cell fusion may influence the balance between these alternative transcription factors. Such a stochastic process may be responsible for the choice of reprogramming direction in ES cell-fibroblast hybrid cells.

## Methods

### Cells and culture conditions

In the present study, ES cells and m5S fibroblasts were used as partners in cell fusion experiments. The murine ES cell line E14Tg2aSc4TP6.3 (briefly, tau-GFP ES cells) carries a deletion in the *hypoxanthine phosphoribosyltransferase* gene and the *pTP6* transgene containing a tau-tagged green fluorescent protein (GFP) and puromycin resistance (Puro) genes^42^. The ES cell line was maintained without a feeder layer.

The fibroblast cell line m5S was established from fibroblasts derived from a male ICR embryo^14^ and obtained from the Japanese Collection of Research Bioresources Cell Bank (Osaka, Japan). The m5S fibroblasts have stable near-diploid karyotype: 42, XO, +15, +der(6)t(6;13)(D;B), and +der(8)t(8;8)(E2;A2). The last rearrangement was used as an easily identified marker chromosome present in all m5S cells. The m5S cells show unlimited proliferation but are sensitive to the contact inhibition of cell division and non-tumorigenicity in *nude* mice^14^. The fibroblasts were grown in DMEM supplemented with 10% fetal bovine serum (Gibco) and 50 μg/ml of penicillin/streptomycin (Gibco).

Different ES cell culture conditions were used in three cell fusion experiments. In the first experiment, the ES cells were grown in standard ES cell medium containing high glucose DMEM (Gibco) and supplemented with 15% ES-cell qualified fetal bovine serum (Gibco), 1X GlutaMAX™-I (Gibco), 1X nonessential amino acids (Gibco), 0.1 mM 2-mercaptoethanol, 1X penicillin/streptomycin and 1,000 U/ml recombinant mouse leukemia inhibitory factor (LIF) (Invitrogen).

In the other two experiments, the ES cells were grown for at least 5 passages prior to cell fusion in ES cell medium supplemented with 2i, small molecule inhibitors PD0325901 (1 µM) and CHIR99021 (3 µM)^16^. Moreover, in one experiment, hybrid cells obtained after fusion were cultured in selective medium supplemented with 2i until the colonies were harvested.

### Cell fusion and production of hybrid cells

Hybrid cells were generated through the fusion of tau-GFP ES cells with m5S fibroblasts as previously described^10^. The next day after cell fusion, ES cell medium containing HAT (100 µM hypoxanthine, 0.4 µM aminopterin, and 16 µM thymidine; Sigma-Aldrich, USA) supplemented with 2 μg/ml of puromycin was used to select hybrid cells. In the second and third cell fusion experiments, the selective medium was supplemented or not with 2i until colony harvesting. The HAT- and puromycin-resistant ES cell-like colonies of hybrid cells were harvested at 14–16 days after fusion, whereas fibroblast-like colonies were harvested at 19–21 days after fusion, reflecting differences in their doubling times.

### Obtaining tetraploid tau-GFP ES cells and m5S cells

Accordingly, 3–5% of tau-GFP ES cells contained a tetraploid set of chromosomes. To isolate these tetraploid ES cells, we seeded individual tau-GFP ES cells onto the wells of a 96-well plate in ES cell medium. Cytogenetic analysis was used to identify clones with tetraploid chromosome sets among dozens of harvested subclones.

The proportion of polyploidy cells (4N) was less than 20% among the m5S cell population^14^. To isolate tetraploid cells from m5S cells, we performed cell sorting according to size using BD FACSAria with BD FACSDiva^TM^ software (BD Biosciences, Canada) at the Collective Flow Cytometry Center of ICG SB RAS, Novosibirsk. The cells with higher FCS-A parameter were subcloned as described above for tau-GFP ES cells. Cytogenetic analysis of the eight subclones resulted in the isolation of clones with tetraploid chromosome sets.

### Karyotype analysis of parental and hybrid cells

The preparation of metaphase chromosomes from diploid and tetraploid m5S fibroblasts and fibroblast-like hybrid cells was performed as previously described^43^, with minor modifications. The metaphase chromosome preparations from diploid and tetraploid tau-GFP ES cells and ES-like hybrid cells were conducted in the following manner: ES cells were cultured in Petri dishes for 1–2 days and for fixation, the cells were treated with 0.1 µg/ml colcemid (ROCHE, Germany) for 1 h. Subsequently, the cells were trypsinized in 0.05% trypsin/EDTA and treated with hypotonic solution (0.25% KCl and 0.2% sodium citrate) directly in the culture plates for 20 min at 37 °C. Next, several drops of fresh Carnoy fixative (methanol:acetic acid 3:1) were added to the Petri dish. The cells were collected, centrifuged and fixed with fresh fixative. Metaphase plates were stained with 4,6-diamidino-2-phenylindole (DAPI; Sigma-Aldrich, USA) and analyzed using a fluorescence microscope (Carl Zeiss Axioplan 2 imaging, Jena, Germany) using a CoolCube1 CCD-camera and processed using ISIS software (*In Situ* Imaging System, MetaSystems GmbH, Altlussheim, Germany) at the Public Center for Microscopy SB RAS, Novosibirsk.

### PCR analysis of microsatellites

Total DNA from tau-GFP ES cells, m5S cells and hybrid cell clones was isolated using TRIzol Reagent (Life Technology, USA) according to manufacturer’s instructions. Microsatellites D13Mit64, D14Mit11, D15Mit226, D17Mit66, D18Mit62, and D19Mit10 were used to detect the parental homologous chromosomes originated from 129 and ICR mice (http://www.informatics.jax.org). The PCR conditions and subsequent electrophoretic analysis of the resulting amplified products were previously described^44^.

### IF analysis of protein markers specific for ES cells and fibroblasts

IF analysis of Oct4, Nanog collagen type I and fibronectin in hybrid cells with both phenotypes was performed as previously described^12^.

### Sequential RNA-DNA, IF-RNA and IF-DNA fluorescence *in situ* hybridization (FISH) analyses

The protocols for these methods have been previously described^45,46^. We used a set of primary antibodies against dimethylated H3K4 (Millipore), trimethylated H3K27 (Millipore), poly- and mono-ubiquitinated proteins (BIOMOL International) and the secondary Alexa Fluor 488-labeled antibodies (Life Technologies) to identify chromatin modifications. An RNA FISH approach with Cot1-labeled DNA as a probe was used to detect actively transcribed and silent chromatin in the nuclei^47^. *Xist* RNA detection on X-chromosomes was performed as previously described^48^. Mouse X and Y repeats used in DNA FISH analysis were prepared as previously described^49^. IF and FISH slides were analyzed on a Nikon Ni-E microscope using NIS-elements software. At least 100 metaphase spreads or approximately 300 nuclei were analyzed for each case. *In vitro* differentiation of the ES-like hybrid clones in the monolayer were performed for 14 days as previously described^50^.

### RNA extraction and RNA-sequencing analysis

Total RNA from tau-GFP ES cells, tau-GFP4N, m5S cells, m5S4N8, and four hybrid clones (two ES-like and two fibroblast-like clones of independent origin) was isolated using TRIzol Reagent (Life Technology, USA) or Aurum Total RNA Mini Kit (Bio-Rad, USA) according to the manufacturer’s instructions. Isolation of RNA was performed in triplicate for each cell line. Multiplex mRNA libraries were obtained using TruSeq RNA sample preparation kits, and the samples were sequenced in three lanes of an Illumina HiSeq. 2000 sequencer (SE, 1 × 51 bp, 6 samples per lane), generating approximately 25 million of reads per sample.

The obtained reads were mapped to the mouse *mm10* genome and analyzed using TopHat, Cufflinks and CumeRbund software according to standard protocols^51,52^. To reduce the number of false-positive results, only genes with FPKM (fragments per kilobase per million) values greater than 10 for at least one of the samples were used to calculate the significance of differences in gene expression levels.

The genes were distributed into categories according to the criteria explained in the *Results* section using homemade Python scripts.

### Single nucleotide polymorphism (SNP) analysis of transcriptome data

SNP calling was performed using SNVer software^53^ and the *mm10* genome as a reference sequence. After calling, all heterozygous SNPs in the parental m5S and tau-GFP transcriptomes were filtered out. The resulting 27,962 SNPs were used to determine the origin of reads in the transcriptome data. Subsequently, the SNP positions were mapped to genes consistent with *mm10* genome annotation according to the Illumina 100 genomes project.

### Data availability

The row sequencing data is available at NCBI (Ref. Number PRJNA394191). The code of homemade scripts used in this study is available at https://github.com/labdevgen/HybridCellsTranscriptome.
